# Posterior Cingulate Cortex-Related Co-Activation Patterns: A Resting State fMRI Study in Propofol-Induced Loss of Consciousness

**DOI:** 10.1371/journal.pone.0100012

**Published:** 2014-06-30

**Authors:** Enrico Amico, Francisco Gomez, Carol Di Perri, Audrey Vanhaudenhuyse, Damien Lesenfants, Pierre Boveroux, Vincent Bonhomme, Jean-François Brichant, Daniele Marinazzo, Steven Laureys

**Affiliations:** 1 Coma Science Group, Cyclotron Research Centre, University of Liège, Liège, Belgium; 2 Faculty of Psychology and Educational Sciences, Department of Data Analysis, Ghent University, Ghent, Belgium; 3 Department of Neurology, University of Liège, Liège, Belgium; 4 Department of Anesthesia and Intensive Care Medicine, CHU Sart Tilman Hospital, University of Liège, Liège, Belgium; 5 Department of Anesthesia and Intensive Care Medicine, CHR Citadelle, University of Liège, Liège, Belgium; 6 Department of Algology and Palliative Care, CHU Sart Tilman Hospital, University of Liège, Liège, Belgium; 7 Department of Neuroradiology, National Neurological Institute C. Mondino, Pavia, Italy; Beijing Normal University, Beijing, China

## Abstract

**Background:**

Recent studies have been shown that functional connectivity of cerebral areas is not a static phenomenon, but exhibits spontaneous fluctuations over time. There is evidence that fluctuating connectivity is an intrinsic phenomenon of brain dynamics that persists during anesthesia. Lately, point process analysis applied on functional data has revealed that much of the information regarding brain connectivity is contained in a fraction of critical time points of a resting state dataset. In the present study we want to extend this methodology for the investigation of resting state fMRI spatial pattern changes during propofol-induced modulation of consciousness, with the aim of extracting new insights on brain networks consciousness-dependent fluctuations.

**Methods:**

Resting-state fMRI volumes on 18 healthy subjects were acquired in four clinical states during propofol injection: wakefulness, sedation, unconsciousness, and recovery. The dataset was reduced to a spatio-temporal point process by selecting time points in the Posterior Cingulate Cortex (PCC) at which the signal is higher than a given threshold (i.e., BOLD intensity above 1 standard deviation). Spatial clustering on the PCC time frames extracted was then performed (number of clusters = 8), to obtain 8 different PCC co-activation patterns (CAPs) for each level of consciousness.

**Results:**

The current analysis shows that the core of the PCC-CAPs throughout consciousness modulation seems to be preserved. Nonetheless, this methodology enables to differentiate region-specific propofol-induced reductions in PCC-CAPs, some of them already present in the functional connectivity literature (e.g., disconnections of the prefrontal cortex, thalamus, auditory cortex), some others new (e.g., reduced co-activation in motor cortex and visual area).

**Conclusion:**

In conclusion, our results indicate that the employed methodology can help in improving and refining the characterization of local functional changes in the brain associated to propofol-induced modulation of consciousness.

## Introduction

Functional magnetic resonance imaging (fMRI) technique has been widely used in the investigation of brain connectivity patterns at rest [1], [2]. Blood-oxygen-level dependent (BOLD) signal activity at rest is organized in correlated spatial patterns, which are called “resting state networks” (i.e. RSNs). These networks have been increasingly investigated and their modulation or disruption has been associated to several pathophysiological conditions [3], [4], together with the importance of these RSNs to provide information about brain dynamic organization, as a complement to structural information.

In particular, in altered states of consciousness it's important to see which functional connections remain unaltered and which ones are modified or disrupted [5], [6]. In this regards, functional connectivity studies of RSNs in induced sedation through anesthesia, have shown widespread changes in fronto-parietal networks, compared with the relative preservation of sensory networks, suggesting a major role of higher-order frontoparietal associative network activity in the loss of consciousness phenomena [7], [8]. Moreover, functional impairment of highly connected frontoparietal areas seems to have greater repercussions on global brain function than on less centrally connected sensorimotor areas [9], [10].

Additionally, recent studies have been shown that functional connectivity of cerebral areas is not a static phenomenon, but exhibits spontaneous fluctuations over time [11]–[13]. Previously, fluctuations of functional connectivity have been thought to reflect changing levels of vigilance, task switching or conscious processing. There is evidence that fluctuating connectivity is an intrinsic phenomenon of brain dynamics that persists even during anesthesia [11]. Fluctuations of functional connectivity within an attention network in macaques have been demonstrated and interpreted as mechanistically important network information [14]. Still, the relationship between changes of consciousness and network dynamics is not understood yet.

Lately, a new approach in exploring functional brain connectivity, using point process analysis, has been proposed by Tagliazucchi et al. [15]. The main idea in this work is that important features of brain functional connectivity at rest can be obtained from BOLD fluctuations, isolating the periods in which the signal crosses some amplitude threshold. In this way the study of the dynamics of a continuous BOLD time series is reduced to the exploration of a discretized one (a point process), defined by time and location of BOLD signal threshold crossings. Through point process analysis, Tagliazucchi and colleagues showed that much of the information regarding a specific RSN is actually contained in a fraction of critical time points (i.e. BOLD signal peaks) of a resting state dataset. This idea has next been adopted by Liu et al. [16], in a study showing that seed-based RSNs extracted from fMRI BOLD signal are averages of multiple distinct spatial co-activations patterns (CAPs) at different time points, and that the analysis of these patterns might provide more fine-grained information on brain functional network organization.

In the present study we want to extend and apply this methodology for the investigation of fMRI resting state spatial pattern changes during propofol-induced modulation of consciousness, with the central aim of extracting new information regarding brain networks consciousness-dependent fluctuations.

## Materials and Methods

### Ethics Statement

The study was approved by the Ethics Committee of the Medical School of the University of Liège (University Hospital, Liège, Belgium). The subjects provided written informed consent to participate in the study.

### Clinical Protocol

The present work is a reanalysis of previous published data [7], [8]. Eighteen healthy right-handed volunteers participated in the study. Subjects fasted for at least 6 h from solids and 2 h from liquids before sedation. During the study and the recovery period, electrocardiogram, blood pressure, pulse oxymetry (SpO2), and breathing frequency were continuously monitored (Magnitude 3150M; Invivo Research, Inc., Orlando,FL). Propofol was infused through an intravenous catheter placed into a vein of the right hand or forearm. An arterial catheter was placed into the left radial artery. Throughout the study, the subjects breathed spontaneously, and additional oxygen (5 l/min) was given through a loosely fitting plastic facemask. The level of consciousness was evaluated clinically throughout the study with the scale used by Ramsay et al. [17].

The subject was asked to strongly squeeze the hand of the investigator. She/he was considered fully awake or to have recovered consciousness if the response to verbal command (squeeze my hand) was clear and strong (Ramsay 2), in sedation if the response to verbal command was clear but slow (Ramsay 3), and in unconsciousness if there was no response to verbal command (Ramsay 5–6). For each consciousness level assessment, Ramsay scale verbal commands were repeated twice. Functional MRI acquisitions consisted of resting-state functional MRI volumes repeated in four clinical states: normal wakefulness (Ramsay 2), sedation (Ramsay 3), unconsciousness (Ramsay 5), and recovery of consciousness (Ramsay 2). The typical scan duration was half an hour in each condition. The number of scans per session (197 functional volumes) was matched in each subject to obtain a similar number of scans in all four clinical states. Functional images were acquired on a 3 Tesla Siemens Allegra scanner (Siemens AG, Munich, Germany; Echo Planar Imaging sequence using 32 slices; repetition time = 2460 ms, echo time = 40 ms, field of view = 220 mm, voxel size = 3.45×3.45×3 mm^3^, and matrix size = 64×64×32).

### fMRI preprocessing

The fMRI data were preprocessed using Statistical Parametric Software (SPM8), performing the typical preprocessing steps of functional connectivity analysis. These steps included motion correction, spatial smoothing (FWHM = 8 mm), temporal filtering with a bandpass filter (0.005 to 0.1 Hz), and the removal of linear and quadratic temporal trends. In addition, the brain-averaged signal, the time series of regions of interest in the white matter and cerebrospinal fluid, and six affine motion parameters were regressed out from the dataset. The fMRI data of each subject was first spatially coregistered to high-resolution anatomical images and then to the 152-brain Montreal Neurological Institute (MNI) space. It has recently been shown that even after standard motion correction, residual head movements can still inflate connectivity measures [18]. In order to evaluate the extent of these residual motion artifact in CAPs, for each subject and for each state of consciousness, we computed the two indices proposed by [18], i.e. Framewise Displacement (FD) and DVARS: D referring to temporal derivative of timecourses, VARS referring to root mean square (RMS) variance over voxels. FD is a scalar quantity that expresses instantaneous head motion, while DVARS is a measure of how much the intensity of a brain image changes in comparison to the previous timepoint [18]. Secondly, we defined as motion corrupted the frames in which FD and DVARS values were both above 0.5 mm for FD and 0.5% Bold for DVARS, as suggested in the same paper. Next, for each state of consciousness, we checked if there were corrupted time frames in our PCC-CAPs, and the percentage of these frames over the whole sample (see also Figure S4). We noticed that the percentage of corrupted time frames in wakefulness was 5% of the total number of frames collected; in sedation 3%; in unconsciousness 8%; in recovery 1%. However, there was no significant difference between CAPs calculated with or without artifact removal. Additionally, the preprocessed fMRI data were resampled to 3×3×3 mm^3^ in the MNI space, and the signal of each voxel was demeaned and normalized by its temporal standard deviation (SD).

### Co-activation patterns construction

**Figure 1:**
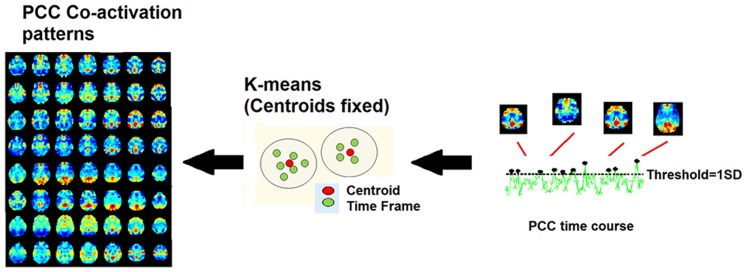
Co-activation patterns (CAPs). The approach is similar to the ones proposed in [16] and [15]. After the extraction of a seed region, in this case Posterior Cingulate Cortex (PCC), a threshold equal to 1 standard deviation(SD) was applied, as to consider only the time points corresponding to peaks in the BOLD signal; next, the spatial maps (namely, time frames), associated with these time points are collected and clusterized using k-means, with a number of clusters fixed to 8. The centroids were kept fixed as well, to allow cluster comparison between the different clinical conditions. The within-cluster time frames were then averaged to obtain 8 spatial PCC-related co-activation patterns. Finally, the computation was iterated over the 4 different states of consciousness (wakefulness, sedation, unconsciousness, recovery), obtaining 8 PCC-related co-activation patterns for each state.

After preprocessing, the dataset was reduced to a spatio-temporal point process [15] by selecting time points in the seed region at which the signal is higher than a given threshold. In this work we used a 6×6×6 mm^3^ cube centered at the posterior cingulate cortex (i.e. PCC, [0, 53, 26] in MNI coordinates, identical to Liu et al. [16]). We chose PCC as seed to study default mode network (DMN) variability during consciousness modulation, since PCC is widely known as a central node in the DMN [19], [20]. CAPs construction can then be summarized in three steps (Fig. 1):

1. First, we collected all the points in the normalized PCC time course where the BOLD signal was above threshold. In our study we fixed the threshold at 1 SD, roughly the 15% of the whole dataset. This percentage did not significantly vary across the four levels of consciousness. For each of these points in the PCC, and for each of the 4 levels of consciousness, we collected the relative spatial maps (Fig. 1). These spatial maps, or time frames [16], represent whole-brain patterns of functional activations correlated to PCC BOLD peaks, previously extracted using this thresholding approach.
2. In order to achieve a spatio-temporal mapping of correlated activity we clustered all the time frames which were significantly co-activated with PCC, in the same way as described in [16]. The sorting of the time frames was performed by K-means clustering, a machine learning classification method able to group unlabeled data into clusters. Once that the desired number of clusters has been fixed, K-means iteratively optimizes the position of the centers in order to minimize the total variance within each cluster [21]. We performed K-means (number of clusters fixed at 8) over all the spatial maps collected to classify the time frames based on their spatial similarity, and then averaging them within-cluster to extract 8 different spatial PCC-related co-activation patterns (i.e. CAPs [16]). Here, since we also aimed to compare different PCC-CAPs between different conditions (i.e. 4 different levels of consciousness), we added an extra step. With the purpose of obtaining a robust benchmark baseline against which to track modifications related to level of consciousness, we first ran k-means clustering over the PCC time frames collected on an independent dataset from the 1000 Functional Connectome Project (FCP, www.nitrc.org/projects/fcon_1000/), which includes wakefulness resting-state functional magnetic resonance imaging (fMRI) collected at multiple sites (247 subjects), as used by Liu and colleagues [16].
3. The eight PCC-CAPs centroids obtained from the clustering of the 1000 Functional Connectome Project dataset (FCP, www.nitrc.org/projects/fcon_1000/) were then kept fixed, and spatial clustering on the PCC time frames extracted from our propofol dataset, for each condition (i.e. wakefulness, sedation, unconsciousness, recovery), was then performed around these centroids, averaging the within-cluster spatial maps to obtain 8 different PCC-CAPs for each level of consciousness. The clustering with centroid fixed allowed us to compare PCC-CAPs between states (i.e. CAP1 in wakefulness with CAP1 in sedation, etc.), and to follow thus the fluctuation of each PCC-CAP over the course of consciousness modulation.

### Statistical analysis

All statistical analyses were carried out using SPM8. For each CAP, individual time frames were entered in a second-level analysis, corresponding to a random effects model in which subjects are considered random variables. These second-level analyses consisted of analyses of variance (repeated measures analysis of variance) with the four clinical conditions as factors: normal wakefulness, sedation, unconsciousness, and recovery of consciousness. The error covariance was not assumed to be independent between regressors, and a correction for non-sphericity was applied. We used one-sided T contrasts, as implemented in Statistical Parametric Mapping software, to test for significant effects in all our analyses. After model estimation, a first T contrast searched for areas co-activated with the PCC during normal wakefulness, sedation, unconsciousness and recovery. Afterwards, in a second analysis a linear one-tailed T contrast was computed for each CAP, searching for a linear relationship between PCC co-activation patterns and the level of consciousness of the subjects across the four conditions (i.e., normal wakefulness, sedation, unconsciousness, and recovery of consciousness, SPM contrast [1.5 −0.5 −1.5 0.5], as previously described in [7]).

It should here be noted that during the recovery of consciousness subjects showed residual plasma propofol levels and lower reaction times scores (table 1 in [7]). Therefore we fixed different SPM contrast values for wakefulness (1.5) and recovery (0.5).

Results were considered significant at p<0.05, corrected for multiple comparisons with False Discovery Rate (FDR [22]), as in [7].

## Results

**Figure 2:**
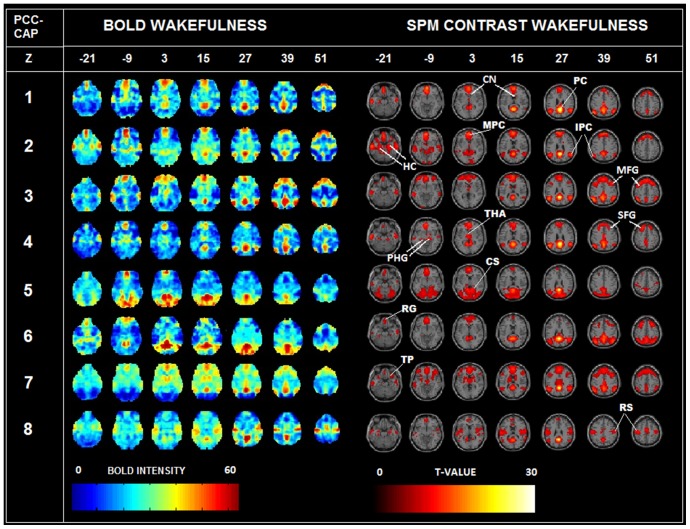
PCC-CAPs in wakefulness. Left: PCC co-activation patterns in wakefulness (colormap normalized by BOLD intensity), current dataset (18 subjects). Note the similarity of these patterns with the ones showed in Fig. 2 of [16]. Right: t-test on the same CAPs in the awake state showing statistically significant PCC co-activations. The seven slices shown in the maps are at Z = −21, −9, 3, 15, 27, 39, 51, respectively. The activation of precuneus (PC) appears in all 8 CAPs (see Z = 27); superior frontal gyrus (SFG) is co-activated in CAPs 1, 2, 3, 4, 6, 7 (Z = 39 and 51); the mesial prefrontal cortex (MPC) in CAPs 1, 2, 3, 4, 5, 6, 7 (Z = 3); rectus gyrus (RG) in CAPs 1, 2, 3,4, 5, 6, 7 (Z = −21); thalamus(THA) in CAPs 4 and 7 (Z = 3); caudate nucleus (CN) in CAPs 1, 3, 7 (Z = 15); temporal pole (TP) in all CAPs (slice Z = −21); hippocampus(HC) in CAPs 2, 4, 6, 8 (Z = −21); parahippocampus gyrus(PHG) in CAPs 2 and 4 (Z = −9); intraparietal cortex(IPC) all CAPs (Z = 27); medial frontal gyrus(MFG) in CAPs 3, 6, 7 (Z = 39); cuneus(CS) in CAPs 4 and 5 (Z = 3); rolandic stripe(RS) in CAPs 5 and 8 (Z = 51). Note how all these region-specific PCC co-activations survive to the t-test (e.g. HC for CAPs 2, 4, 6, 8; MFG for CAPs 3, 6, 7; PHG for CAPs 2, 4 etc.)

We studied PCC-CAPs in our 18 subjects fMRI resting state dataset, for all the 4 different states of consciousness acquired, i.e. normal wakefulness, sedation, unconsciousness, recovery (see **Material and Methods** section for details). Fig. 2 shows the CAPs obtained in our study, before and after t-contrast on the significant co-activations. These spatial patterns are well reproduced in our smaller cohort (compared to the one obtained from the 1000 Functional Connectome Project dataset, depicted in Fig. 2 of [16]), and they also appear to be statistically significant(see Legend Fig. 2 for details).

**Figure 3:**
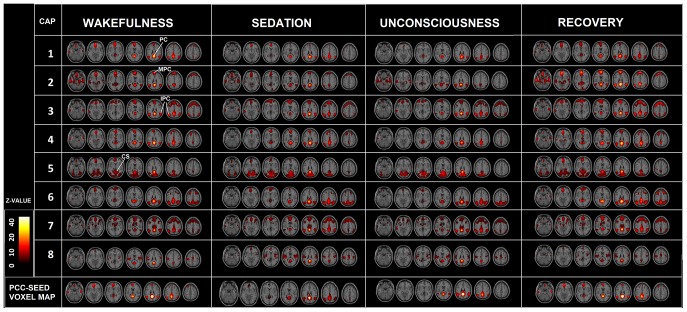
CAPs at different levels of consciousness. Co-activation patterns (t-contrast) in wakefulness (left), sedation (middle-left), unconsciousness (middle-right), recovery (right), corrected at FDR p<0.05. Lower panel shows PCC seed-voxel correlation maps for each state. The seven slices shown in the maps are at Z = −21, −9, 3, 15, 27, 39, 51, respectively. Spatial patterns in wakefulness and recovery do not significantly differ from each other. Precuneal (PC) activations are preserved in all CAPs in all conditions (Z = 27 and Z = 39); mesial prefrontal (MPC) cortical activity is preserved in CAPs 1, 2, 3, 4 and 7 during wakefulness, sedation and recovery (Z = 27); intraparietal cortex (IPC) activation is preserved in CAPs 1, 2, 3, 4, 6 and 7 in all conditions (Z = 39); cuneus(CS) in CAPs 4 and 5 in all conditions (Z = 3). Note that PCC-CAPs add spatial region-specific information to the network characterization across the different stages of consciousness, when compared to the equivalent PCC seed-based contrast (last row), which exhibit a prominent drop of frontal activation during sedation and unconsciousness.

Fig. 3 illustrates significant PCC co-activations in wakefulness, sedation, unconsciousness and recovery. The coarse core of the patterns throughout the consciousness modulation seem to be preserved (see Legend Fig. 3 for details). The comparison with the common seed-voxel analysis (bottom) helps to understand the advantage of this methodology, that is the ability to differentiate spatially the activation, adding fine grained information [16].

**Figure 4:**
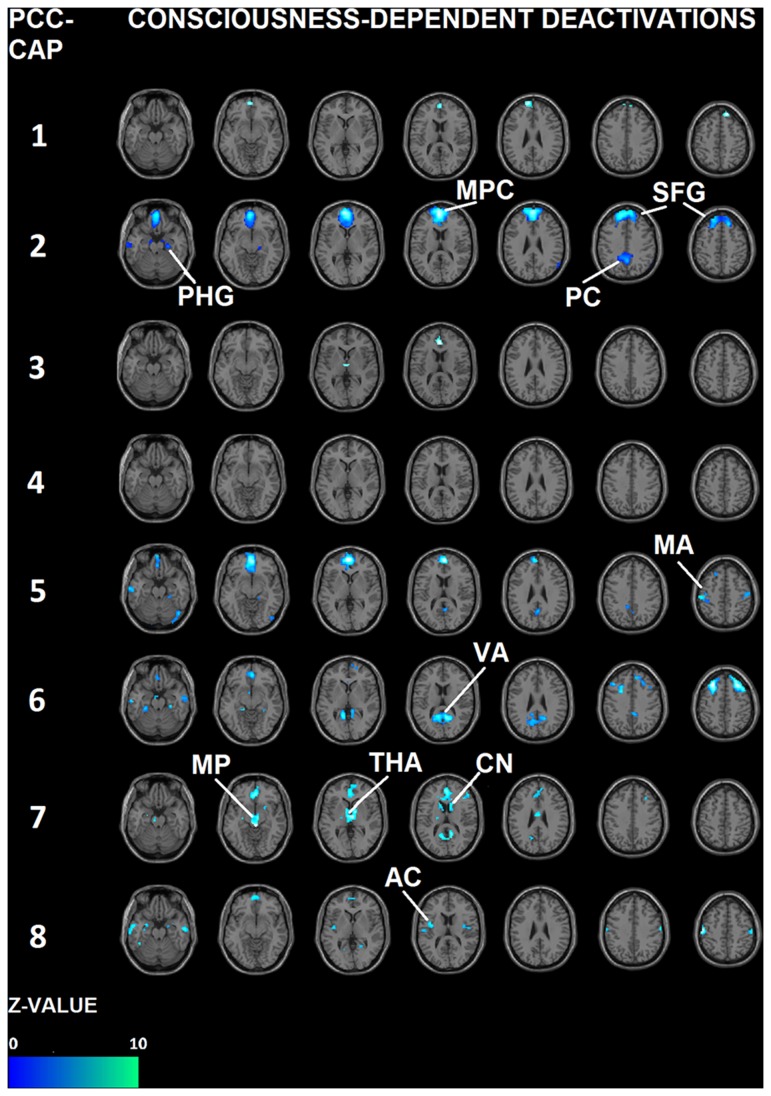
Decreases in CAPs. This figure shows the local decreases in co-activation from wakefulness to unconsciousness, using the same t-contrast as in [7]. All the images report contrast which are significant at p<0.05, FDR corrected. The seven slices shown in the maps are at Z = −21, −9, 3, 15, 27, 39, 51, respectively. CAPs consciousness-dependent deactivations appear in mesial prefrontal cortex (MPC), CAPs 1, 2, 5 (see Z = 15); superior fontal gyrus (SFG) in CAP 2 (Z = 39 and 51); thalamus (THA) in CAPs 3 and 7 (Z = 3); mesencephalon (MP) in CAP 7 (Z = −9); motor area (MA) in CAP 5 and CAP 8 (Z = 51); parahippocampal gyrus (PHG) in CAPs 2, 5, 6 (Z = −21); caudate nucleus (CN) in CAP 7 (Z = 15); visual area (VA) in CAP 6 (Z = 15); auditory cortex (AC) in CAP 8 (Z = 15) and precuneus (PC) in CAP 2 (Z = 39). For details see also Fig. 5 and Table 1.

**Figure 5:**
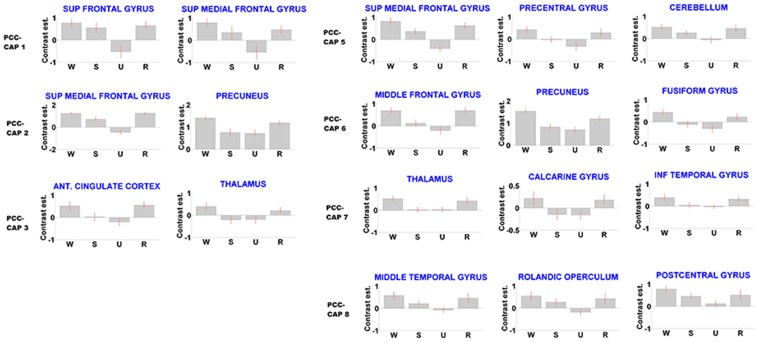
Region specific consciousness modulation in CAPs. Parameters estimates for the t-contrast employed, identical as in [7], for some indicative regions where PCC-related activation correlates with propofol-induced changes in consciousness. (mean ± SE; x axis labels: W = wakefulness; S = sedation; U = unconsciousness; R = recovery). Note how PCC co-activation in these regions significantly follows consciousness modulation (see also Table 1).

Preserved CAPs aside, some regions are no longer co-activated with the PCC in states of propofol-reduced consciousness. As to better quantify this phenomenon we decided to use a contrast that correlates with levels of consciousness. Fig. 4 illustrates the regions in each PCC-CAP where activity follows consciousness modulation. Interestingly, results show several region specific drops in CAPs activation that we are able to differentiate thanks to the employed methodology, some of them already shown in literature, some others not. In Fig. 4, CAP 1, 2 5 and 7 show drop in the prefrontal cortex, CAP 3 and 7 isolates the thalamic drop, the auditory and motor cortex decreases come up in CAP 8 and 5, drop of the visual area in CAP 6 (see also Fig. 5 and Table 1 for details).

**Table 1:** Stereotactic coordinates of peak voxels in clusters of PCC-CAPs showing a linear correlation with propofol-induced changes in consciousness.

CAP 1	x	y	z	Z-value	FDR-p
Right Superior Frontal Gyrus	18	42	48	5.41	0.002
Left Superior Medial Frontal Gyrus	−9	60	30	5.12	0.002
Left Mid Orbital Gyrus	0	57	−9	4.65	0.004
Right Superior Medial Frontal Gyrus	9	57	36	4.06	0.016

CAP 2	x	y	z	Z-value	FDR-p
Left Superior Medial Frontal Gyrus	−3	60	9	6.55	<0.001
Left Superior Frontal Gyrus	−15	60	24	6.55	<0.001
Right Precuneus	3	−54	36	5.54	<0.001
Left Middle Cingulate Cortex	0	−36	39	3.09	0.011
Right ParaHippocampal Gyrus	24	−21	−18	4.47	<0.001
Right Angular Gyrus	60	−63	33	3.97	<0.001
Left Middle Temporal Gyrus	−63	−15	−24	3.38	0.005

CAP 3	x	y	z	Z-value	FDR-p
Left Anterior Cingulate Cortex	0	39	15	5.23	0.003
Thalamus	0	−12	0	4.48	0.012

CAP 5	x	y	z	Z-value	FDR-p
Right Superior Medial Frontal Gyrus	3	57	12	6.55	<0.001
Left Superior Medial Frontal Gyrus	0	57	3	6.55	<0.001
Left Precentral Gyrus	−39	−18	60	5.47	<0.001
Right Cerebellum	27	−33	−33	5.053	<0.001
Hippocampus	15	−33	−9	3.47	0.011
Right Precuneus	3	−57	21	4.83	<0.001
Left Middle Temporal Gyrus	−66	−15	−21	4.75	<0.001
Right Inferior Occipital Gyrus	48	−81	−6	4.03	0.002
Left Superior Frontal Gyrus	−21	30	48	4.51	<0.001
Right Postcentral Gyrus	57	−15	48	4.28	<0.001
Right Precentral Gyrus	48	−15	60	3.64	0.006
Left Precuneus	−9	−45	39	3.61	0.007

CAP 6	x	y	z	Z-value	FDR-p
Left Middle Frontal Gyrus	−24	30	54	7.52	<0.001
Right Superior Frontal Gyrus	27	27	54	7.11	<0.001
Right Middle Frontal Gyrus	45	18	48	5.68	<0.001
Right Precuneus	9	−54	15	6.12	<0.001
Left Hippocampus	−15	−33	−6	5.09	<0.001
Left Middle Temporal Gyrus	−57	−18	−24	5.55	<0.001
Right Fusiform Gyrus	30	−27	−21	4.71	<0.001
Right ParaHippocampal Gyrus	30	−36	−9	3.69	0.005

CAP 7	x	y	z	Z-value	FDR-p
Left Thalamus	6	−12	9	5,48	<0.001
Right Thalamus	6	−12	0	5,48	<0.001
Left Anterior Cingulate Cortex	0	48	9	5,25	<0.001
Left Precuneus	−15	−60	21	4,67	0,001
Right Precuneus	12	−51	15	4,43	0,002
Right Calcarine Gyrus	12	−60	15	4,37	0,002
Right Middle Frontal Gyrus	33	30	45	4,19	0,003
Right Superior Frontal Gyrus	24	33	54	3,61	0,012
Right Inferior Temporal Gyrus	54	3	−39	3,93	0,006
Left Middle Cingulate Cortex	0	−33	48	3,86	0,007
Hippocampus	−24	−9	−21	3,84	0,007
Right Inferior Frontal Gyrus	48	39	15	3,74	0,009
Right Inferior Parietal Lobule	51	−51	57	3,47	0,016

CAP 8	x	y	z	Z-value	FDR-p
Left Postcentral Gyrus	−54	−18	54	6,58	<0.001
Right Postcentral Gyrus	63	−12	42	4,86	0,001
Right Precentral Gyrus	48	−6	60	3,91	0,011
Right Middle Temporal Gyrus	60	−12	−24	4,76	0,002
Left Superior Medial Frontal Gyrus	0	57	6	3,82	0,013
Left Anterior Cingulate Cortex	−12	45	0	3,65	0,018
Left Middle Temporal Gyrus	−57	−6	−18	4,43	0,005
Left Rolandic Operculum	−39	0	15	4,32	0,005
Left Insula Lobe	−42	−9	3	3,84	0,012
Right Rolandic Operculum	48	−12	21	4,15	0,007
Left Amygdala	−27	−3	−24	3,95	0,011
Right Hippocampus	30	−3	−24	3,95	0,011

## Discussion

With the aim to investigate changes in global and local brain activity, in this study we assessed propofol-induced changes in PCC co-activation functional patterns, during wakefulness, sedation, unconsciousness, and wakefulness recovery. Our results contribute to a growing literature addressing the changes in functional connectivity accompanying the loss of consciousness [23]–[26]. The approach proposed, based on the clustering of instantaneous PCC-related spatial maps, helps in the refinement and the differentiation in the spatial modulation of the default mode network when switching from wakefulness to unconsciousness.

Two key aspects of the methodology proposed in [16], and extended here, are worth discussing. One is the choice of the threshold, that is a crucial step for the construction of a spatio-temporal point process from BOLD time series. We decided to fix our threshold at 1 SD, as in [15], rather than address the threshold in terms of time frame similarity with the seed-based correlation map (see Fig. 1B in [16]). The hypothesis behind this choice is that BOLD signal point processing can help in the spatial characterization of functional connectivity patterns, better than standard correlation analysis (see also Figure S2 and S3). This data-driven evidence is independent of the assumption that BOLD signal peaks may or may not reflect cortical activations [15], [27]. In this study we simply focused on spatial clustering of point processed BOLD time series, which allowed us to better characterize functional spatial interaction with the PCC during modulation of consciousness. On the other hand, the employed point process methodology is not purely dominated by hemodynamics. Indeed, co-activations patterns obtained after deconvolving the hemodynamic response function (HRF) at rest as in [27], are not qualitatively different from the ones presented in Fig. 2 (see also Figure S1).

Furthermore, the results from our PCC coactivation patterns seem in line with the ones obtained by Liu [16], in a different cohort and with a different choice of the threshold. This comes out on the side of point process methodology and of the importance of these BOLD signal peaks in spatial differentiation of network co-activations. Also, the use of clustering with centroid fixed, that is new with respect to the approach proposed in [16], can be an useful benchmark for statistical comparison of functional changing patterns between conditions (conscious states in this case, but it could also be used in pathological conditions, etc.).

A primary result is that the core of PCC-CAPs is preserved in anesthesia (Fig. 3). This is in line with other studies [7], [14], where, using standard seed-based functional connectivity, the preservation of a core network of correlated regions independent on the level of consciousness was confirmed. The origin of these functional networks could be related to the fixed structural connection present in the awake brain, albeit anesthetized. This partial preservation of functional connectivity in the absence of consciousness has been suggested to possibly reflect preserved anatomical connections dissociated from higher cognitive functions [28].

Also, this approach adds new information on region specific drops in connectivity between the seeds in the DMN area and whole brain connectivity, correlating with levels of consciousness (Fig. 4 and Fig. 5): prefrontal drops (CAP 1, 2, 5, 7) are in line with previous findings in the field, showing widespread changes in prefrontal connectivity [7], [9], [10]: at the cortical level, hypnotic anesthetic agents have traditionally been considered to decrease activity in a widespread bilateral frontoparietal network. The primary action of hypnotic anesthetic agents would be to functionally disconnect different parts of the cortex, which would probably impair the ability to integrate information [8], [23], [29]. A recent electroencephalography study similarly suggests the presence of some anterior-posterior functional uncoupling in the brain, during anesthesia-induced loss of consciousness [30].

The disconnection of the thalamic area, already noted in [31], [32], is highlighted in CAP 3 and 7. Using positron emission tomography, thalamic metabolism has been shown to decrease significantly during anesthesia-induced unconsciousness [24]. Furthermore, a model has been suggested in which the thalamus orchestrates the commonly observed increased and coherent alpha frequency activity in the frontal cortex during propofol-induced unconsciousness. This steady thalamic alpha rhythm could impede conduction and thus responsiveness to external stimuli [33].

Decrease of activation in the visual area (CAP 6) during anesthesia has previously been studied in monkeys [34], [35], where it has been shown how local and global processing in the visual area might depend gradually on the depth of anesthesia. It may also critically depend on information integration mechanisms that function properly only in the awake and perceiving animal [34]. Here, this disconnection is shown in fMRI resting state on humans: thus, this approach seem to enlighten instantaneous spatial connectivity changes between DMN and other external areas, unlikely to be seen with other commonly used correlation analysis (e.g. seed-based functional connectivity).

Similarly, the PCC-related primary motor disconnection is pointed out here (CAP 5 and 8) on fMRI resting state data. These results are in line with previous transcranial magnetic stimulation (TMS) and electroencephalographic (EEG) studies, that indicate intracortical inhibition of central motor circuitry during incremental suppression by a potentiator of GABA agonist (propofol) in a dose-dependent manner [36], [37]. The decrease in auditory cortex PCC-coactivation (CAP8) is in agreement with previous findings, in animals [38] and humans [39]. Our finding of decreased auditory cortex co-activation could be related to the hypothesis that propofol bilaterally attenuates the auditory-induced BOLD signal activation of the auditory cortex in a dose-dependent manner [40]. However, it should be noted that activation studies and resting state acquisitions offer different assessments of the underlying neurophysiological activity, in response to external stimulation or during resting conditions, respectively.

A limitation to this approach is the choice of the seed, that needs to be based on a strong priori hypothesis: whole brain connectivity analysis can improve research in this direction. Finally, since it has recently been shown that EEG directional connectivity shows characteristic changes during propofol-induced unconsciousness [41], the nature of BOLD peaks and their correlation with cortical activity needs to be explored using combined fMRI-EEG recordings.

In conclusion, our result show that functional changes in the brain associated to propofol-induced modulation of consciousness can be efficiently revealed by tracking the patterns of co-activation in the Posterior Cingulate Cortex, an area with a central role in the dynamical connectivity at rest. This methodology, based on point process analysis, can help in refining the characterization of local functional disconnections following the partial or total loss of consciousness.

## Supporting Information

Figure S1 **CAPs from BOLD and deconvolved BOLD signal.** To avoid the possibility that the PCC-coactivation patterns were only due to different hemodynamic response functions in the different areas of the brain, we applied the approach proposed in Wu et al. [27], where point process is used to deconvolve the HRF at rest from the BOLD signal. As shown above, the CAPs obtained from the BOLD signal reported in the manuscript are confirmed when obtained from the deconvolved BOLD, suggesting a connection between spatial functional co-activations and neuronal brain response.
(TIF)

Figure S2 **CAPs from negative BOLD peaks.** CAPs obtained using positive threshold crossing (left) and negative threshold crossing (right), during wake resting state (CAPs colormap in absolute Z-value, to make patterns comparable). After the clustering, the specificity of the spatial patterns obtained using positive peaks in BOLD is not reproducible using negative peaks; positive BOLD peaks allow to reconstruct a richer variety of patterns.
(TIF)

Figure S3 **Sliding window correlation vs CAPs.** PCC co-activation patterns in wakefulness (left) compared to the 8 patterns obtained after spatial clustering of the N PCC-correlation maps computed using sliding window correlation, with window size varying from 5 time points (i.e. 12 s window, TR = 2.46 s) to 20 time points (i.e. 50 s window). Note that the region-specific patterns obtained with point process on the BOLD peaks are not recovered by using sliding window correlation. This approach seems to add more refined information in the spatial differentiation of functional networks.
(TIF)

Figure S4 **Motion correction in CAPs.** Example of the procedure discussed in **Materials and Methods**, for one subject, for each level of consciousness (i.e. wakefulness, sedation, unconsciousness, recovery). In order to evaluate the extent of these residual motion artifact in CAPs, for each subject and for each state of consciousness, we computed the two indices proposed by [18], i.e. Framewise Displacement (FD) and DVARS. FD is a scalar quantity that expresses instantaneous head motion, while DVARS is a measure of how much the intensity of a brain image changes in comparison to the previous time point [18]. Secondly, we defined as motion corrupted the frames (ArtFrames in the figure) in which FD and DVARS values were both above 0.5 mm for FD and 0.5.
(TIF)
