# Supplementary figures and images for: Posterior Cingulate Cortex-Related Co-Activation Patterns: A Resting State fMRI Study in Propofol-Induced Loss of Consciousness
Source: PLoS One. 2014 Jun 30;9(6):e100012. doi: 10.1371/journal.pone.0100012 (PMC4076184; doi:10.1371/journal.pone.0100012)

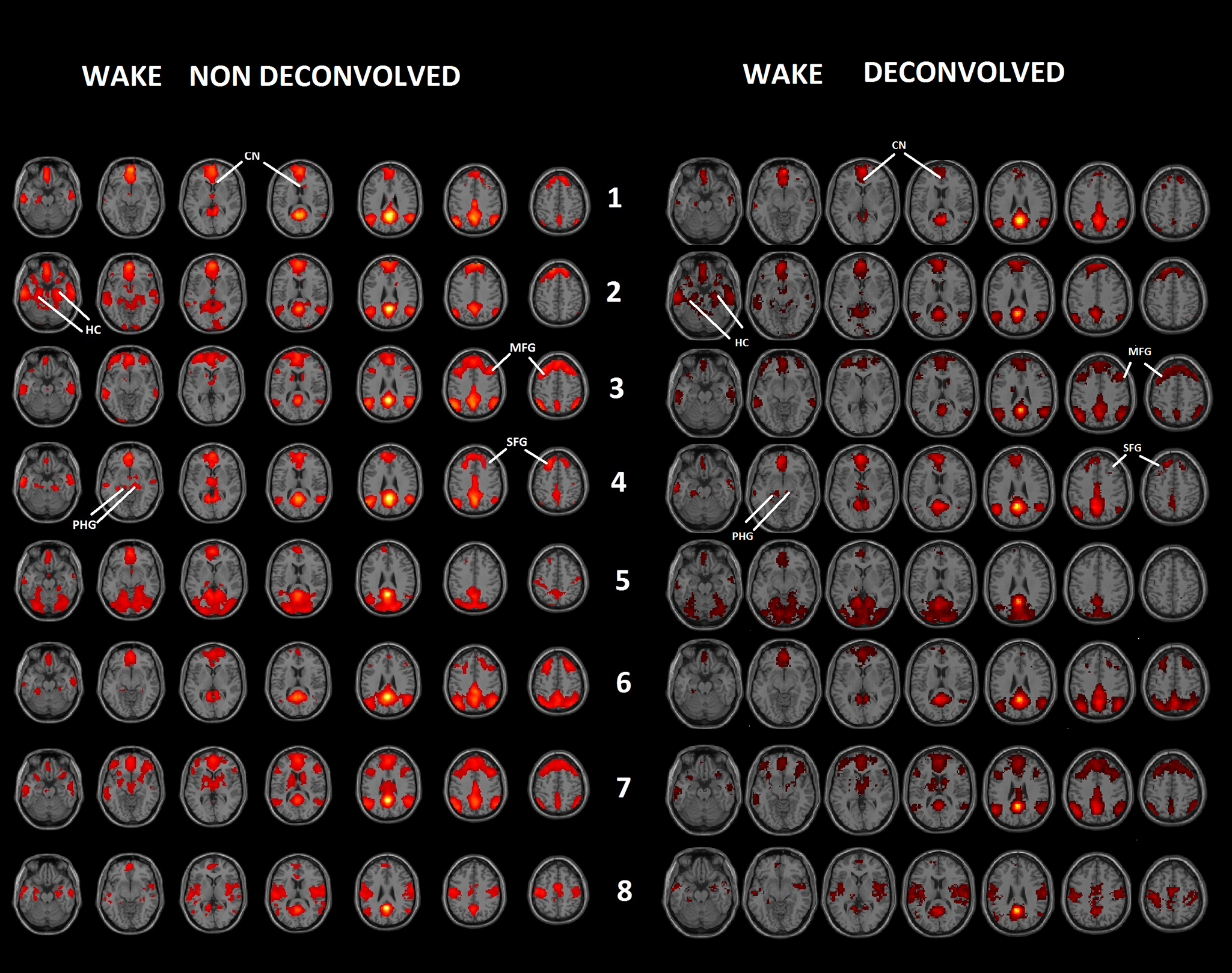

Supplement: Figure S1 — CAPs from BOLD and deconvolved BOLD signal. To avoid the possibility that the PCC-coactivation patterns were only due to different hemodynamic response functions in the different areas of the brain, we applied the approach proposed in Wu et al. [27], where point process is used to deconvolve the HRF at rest from the BOLD signal. As shown above, the CAPs obtained from the BOLD signal reported in the manuscript are confirmed when obtained from the deconvolved BOLD, suggesting a connection between spatial functional co-activations and neuronal brain response. (TIF) [file pone.0100012.s001.tif]

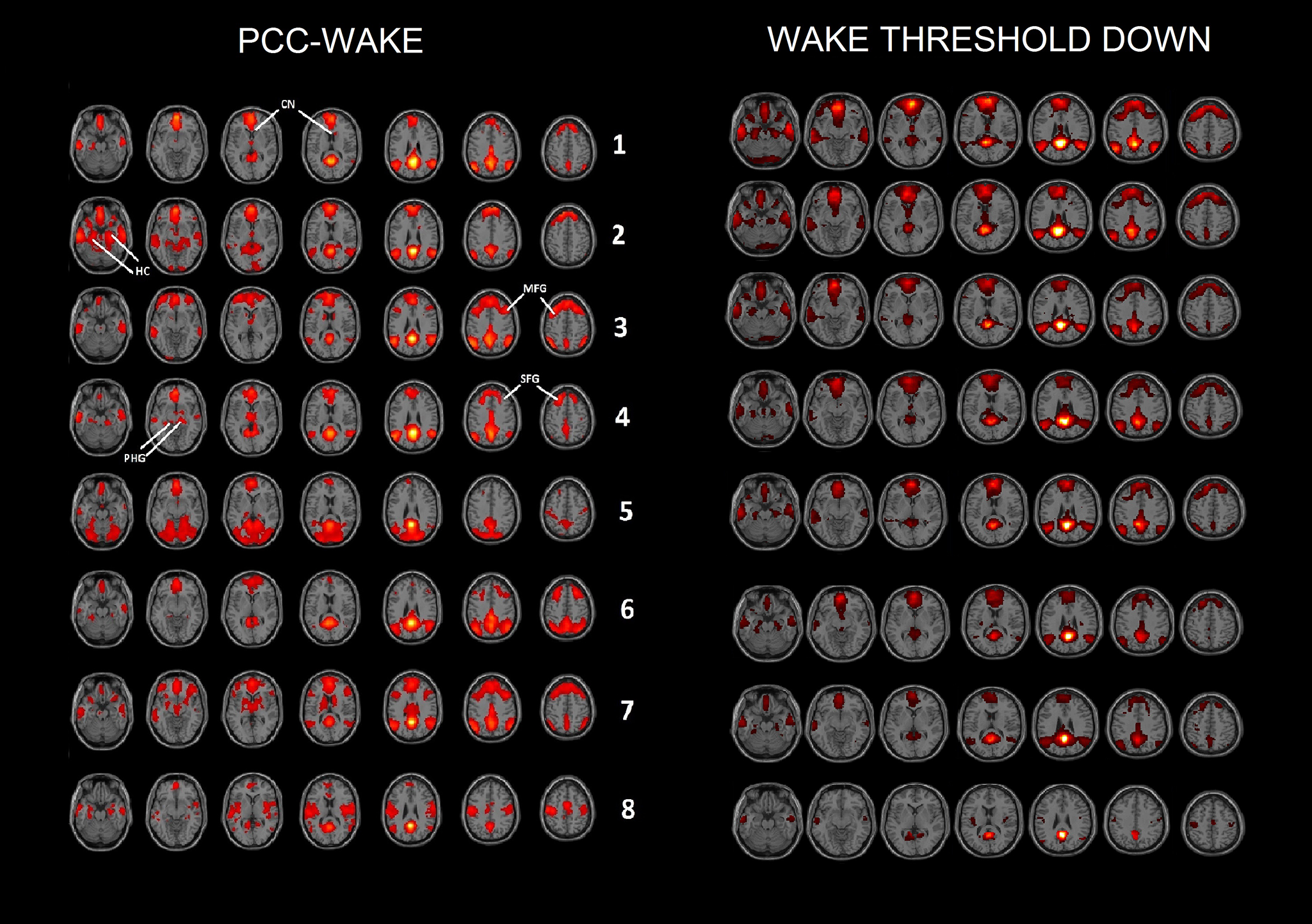

Supplement: Figure S2 — CAPs from negative BOLD peaks. CAPs obtained using positive threshold crossing (left) and negative threshold crossing (right), during wake resting state (CAPs colormap in absolute Z-value, to make patterns comparable). After the clustering, the specificity of the spatial patterns obtained using positive peaks in BOLD is not reproducible using negative peaks; positive BOLD peaks allow to reconstruct a richer variety of patterns. (TIF) [file pone.0100012.s002.tif]

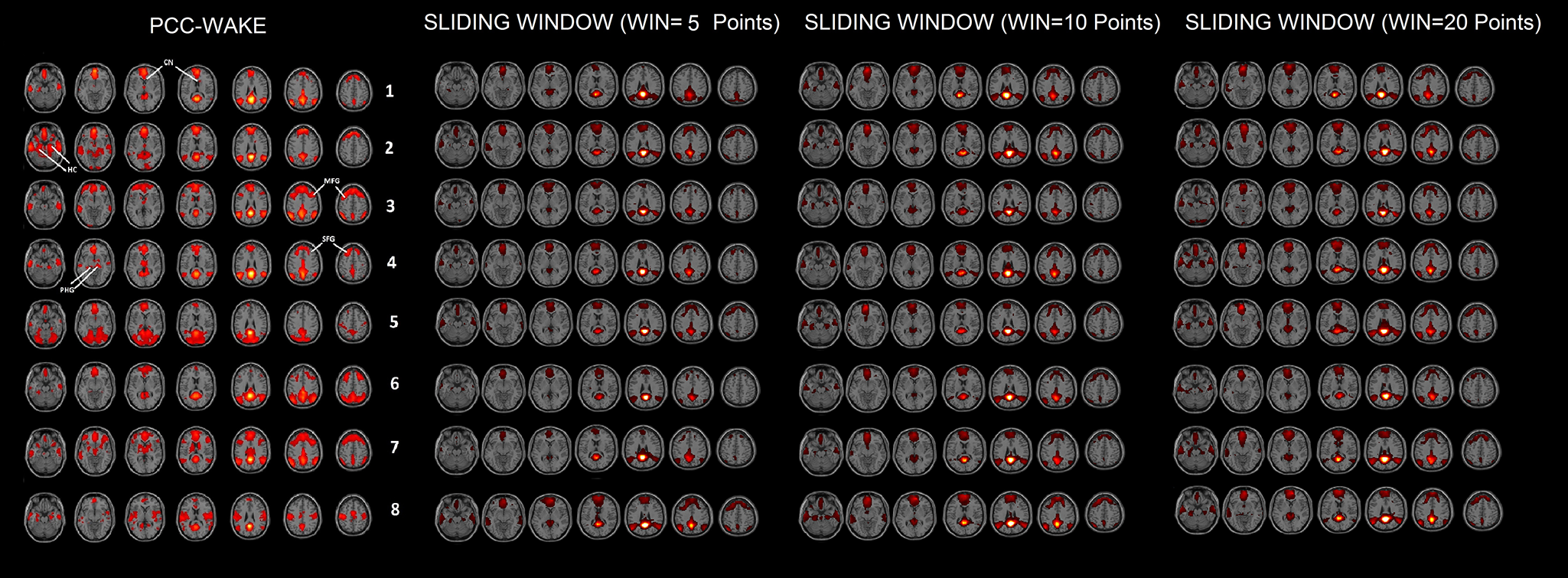

Supplement: Figure S3 — Sliding window correlation vs CAPs. PCC co-activation patterns in wakefulness (left) compared to the 8 patterns obtained after spatial clustering of the N PCC-correlation maps computed using sliding window correlation, with window size varying from 5 time points (i.e. 12 s window, TR = 2.46 s) to 20 time points (i.e. 50 s window). Note that the region-specific patterns obtained with point process on the BOLD peaks are not recovered by using sliding window correlation. This approach seems to add more refined information in the spatial differentiation of functional networks. (TIF) [file pone.0100012.s003.tif]

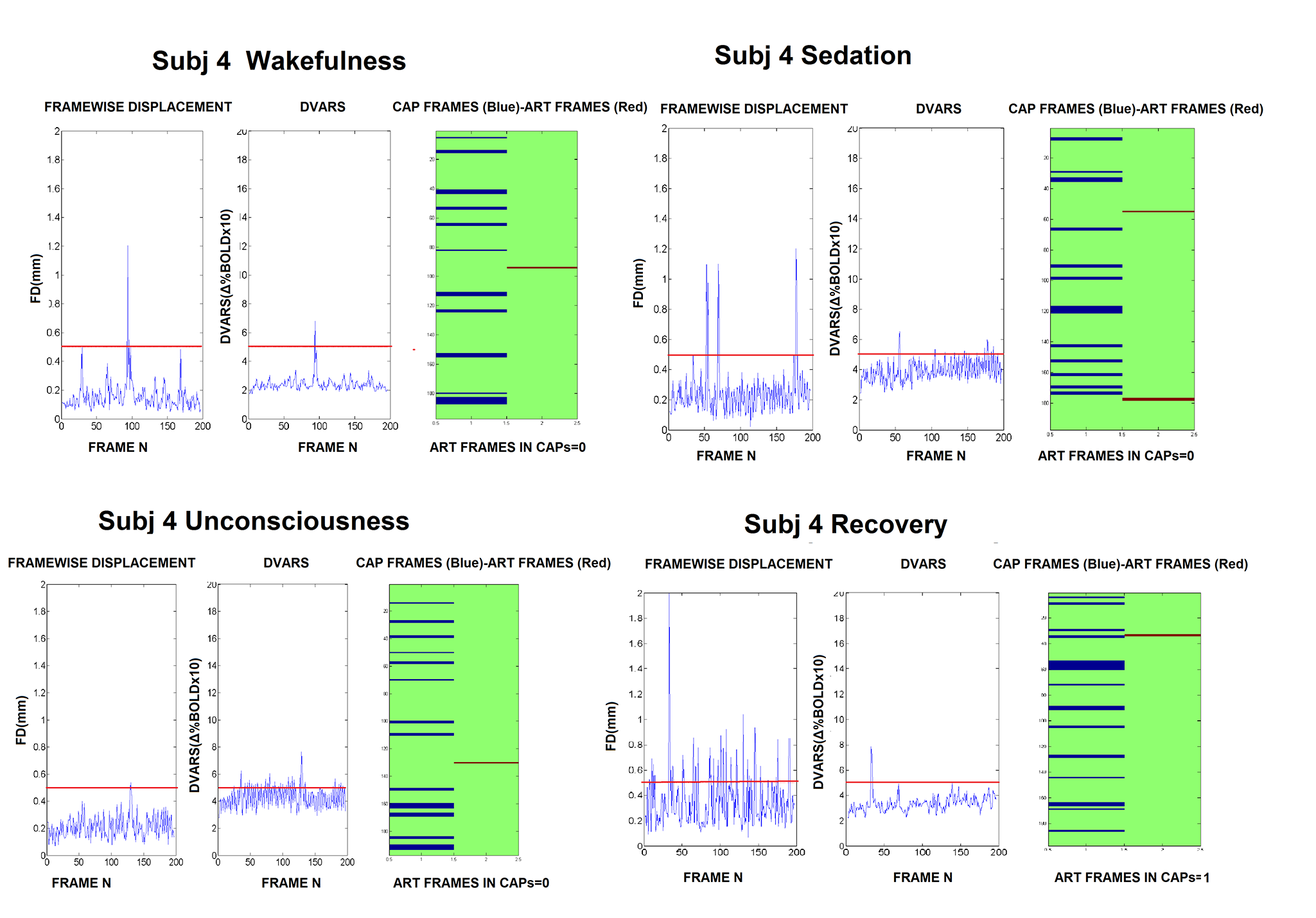

Supplement: Figure S4 — Motion correction in CAPs. Example of the procedure discussed in Materials and Methods , for one subject, for each level of consciousness (i.e. wakefulness, sedation, unconsciousness, recovery). In order to evaluate the extent of these residual motion artifact in CAPs, for each subject and for each state of consciousness, we computed the two indices proposed by [18], i.e. Framewise Displacement (FD) and DVARS. FD is a scalar quantity that expresses instantaneous head motion, while DVARS is a measure of how much the intensity of a brain image changes in comparison to the previous time point [18]. Secondly, we defined as motion corrupted the frames (ArtFrames in the figure) in which FD and DVARS values were both above 0.5 mm for FD and 0.5. (TIF) [file pone.0100012.s004.tif]
